# Lessons on neurodegeneration and aging from the Lagoon of Venice: the marine invertebrate *Botryllus schlosseri*

**DOI:** 10.1093/braincomms/fcae257

**Published:** 2024-09-25

**Authors:** Tommaso Bocci, Chiara Anselmi, Federico La Torre, Emanuela De Lisa, Giacomo Sabbadin, Matteo Guidetti, Natale Maiorana, Alberto Priori, Lucia Manni

**Affiliations:** ‘Aldo Ravelli’ Center for Neurotechnology and Experimental Brain Therapeutics, Department of Health Sciences, University of Milan, 20142 Milan, Italy; Clinical Neurology Unit, ‘Azienda Socio-Sanitaria Territoriale Santi Paolo E Carlo’, Department of Health Sciences, University of Milan, 20142 Milan, Italy; Hopkins Marine Station, Institute for Stem Cell Biology and Regenerative Medicine, Stanford University, Pacific Grove, CA 93950, USA; Institute for Stem Cell Biology and Regenerative Medicine, Stanford University School of Medicine, Stanford, CA 94305, USA; Wu Tsai Neurosciences Institute, Stanford University, Stanford, CA 94305, USA; Dipartimento di Biologia, Università degli Studi di Padova, 35131 Padua, Italy; Dipartimento di Biologia, Università degli Studi di Padova, 35131 Padua, Italy; Dipartimento di Biologia, Università degli Studi di Padova, 35131 Padua, Italy; ‘Aldo Ravelli’ Center for Neurotechnology and Experimental Brain Therapeutics, Department of Health Sciences, University of Milan, 20142 Milan, Italy; Clinical Neurology Unit, ‘Azienda Socio-Sanitaria Territoriale Santi Paolo E Carlo’, Department of Health Sciences, University of Milan, 20142 Milan, Italy; ‘Aldo Ravelli’ Center for Neurotechnology and Experimental Brain Therapeutics, Department of Health Sciences, University of Milan, 20142 Milan, Italy; Clinical Neurology Unit, ‘Azienda Socio-Sanitaria Territoriale Santi Paolo E Carlo’, Department of Health Sciences, University of Milan, 20142 Milan, Italy; ‘Aldo Ravelli’ Center for Neurotechnology and Experimental Brain Therapeutics, Department of Health Sciences, University of Milan, 20142 Milan, Italy; Clinical Neurology Unit, ‘Azienda Socio-Sanitaria Territoriale Santi Paolo E Carlo’, Department of Health Sciences, University of Milan, 20142 Milan, Italy; Dipartimento di Biologia, Università degli Studi di Padova, 35131 Padua, Italy

## Abstract

In this work, the authors proposed a novel and interesting animal model for studying human neurodegenerative diseases, *Botryllus schlosseri*, a small invertebrate inhabiting temperate seas worldwide, which shares remarkable similarities with mammals in the expression of genes involved in pathological aging.

Aging is a critical and necessary phase of our existence, and the circle of death and life dominates and drives all our daily choices. However, as stated by Saint Paul in his first Letter to the Corinthians, ‘Death will be the last enemy we’ll defeat’ (Letter to Corinthians, 15:26), we are not any closer to understanding secrets of death and life. In recent decades, as the life expectancy increased, pathological aging gained more and more importance.

Since its first description in 1817,^1^ Parkinson’s disease gained growing attention as one of the most common human neurodegenerative disorders, probably strictly related to environmental factors accelerating the process of aging. Whereas the aetiology of Parkinson’s disease remains unknown, some of its pathophysiological mechanisms have been increasingly elucidated.^2^ As for Alzheimer’s disease, neurodegenerative disorders are now thought to represent ‘proteinopathies’ (or, according to some recent definitions, ‘proteinopenias’), in which a critical step of neurodegeneration itself is the aggregation of misfolded proteins, thus driving both impaired lysosomal degradation and defective axonal transport.^3,4^ The epidemiology of Alzheimer’s disease is interlaced with that of all-cause dementia, and it is expected to rise from 50 million people in 2010 to 113 million in 2050.^5^ The estimated prevalence of Parkinson’s disease in industrialized countries is ∼1% in people over 60.^6^

Despite recent advances in understanding the pathophysiological mechanisms of Parkinson’s disease and Alzheimer’s disease, either pharmacological or non-pharmacological therapies remain exclusively symptomatic, without any evidence to substantially modify disease progression or its mechanisms. So, is the elixir of life merely a myth? Given the current state of knowledge, it seems that way. However, a ray of hope may come from a little marine invertebrate, formally named *Botryllus schlosseri*, forming colonies on stones, ropes and any marine hard substrate in temperate sea water. This cosmopolitan colonial chordate is found in shallow water around the word, including the Venice Lagoon where it has been harvested and cultured while serving a focus of Padova University research programme for the last 60 years (Fig. 1). A single animal is only a couple of millimetres long, but a colony can be composed of thousands of individuals. Despite its simplicity and phenotype diversity with respect to humans, *Botryllus* is a member of a clade, the tunicates, considered to be the sister group of vertebrates. Indeed, it exhibits developmental mechanisms that have intriguing commonalities with vertebrate development, also at nervous system level.

**Figure 1:**
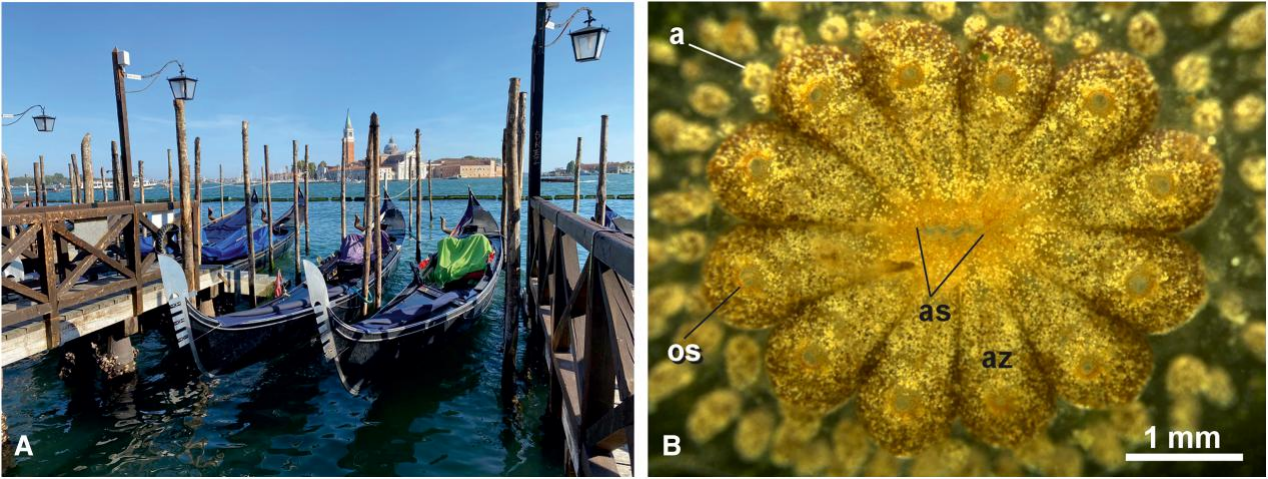
**The Venice Lagoon of Venice and *B. schlosseri*.** (**A**) View of the Lagoon of Venice, seen from Piazza San Marco in Venice towards the Church of Redentore (by Palladio, 1570). (**B**) *Botryllus schlosseri* colony in dorsal view. Adult zooids (az) are aggregated in star-shaped systems. Each zooid has its own oral siphon (os) from which seawater enters the body for feeding and respiration whereas the atrial siphon (as) is common to all the zooids and is located at the system centre. A transparent tunic embeds the zooids and the colonial circulatory system, represented by vessels and their terminal ampullae (a). Adult zooids cover their buds, so that they are not commonly visible in dorsal view. Scale bar: 1 mm.

Not only does this creature share a high genomic homology with mammals,^7^ but it also provides a unique research opportunity thanks to the peculiarity of its life cycle. Indeed, *Botryllus* engages in both sexual and asexual reproduction giving rise, respectively, to a swimming tadpole larva, destined to metamorphosis in the sessile individual founding a new colony, and to individuals called zooids. The latter undergo a cyclical budding process (occurring weekly at 18°C in laboratory conditions) that produces genetically identical zooids allowing colonies to grow quickly. Colonies can be fragmented creating clones of genetically identical zooids (twin colonies), to be used experimentally and as control. What makes the *Botryllus* life cycle special is a recurrent life phase, called takeover, marked by the development of multiple buds and the eventual degeneration of adult zooids: colonies weekly rejuvenate, thanks to the appearance of new buds and the simultaneous death of the old individuals. This degeneration involves all the adult organs, including the brains. Each brain begins to develop in the buds, ultimately reaching a mature stage of ∼900 cells in the adults before undergoing degeneration, resulting in a loss of neurons and associated behavioural function.^8^ Hence, overall, *Botryllus* is a fast-aging model, allowing to study neurodegeneration repeatedly (every week in lab), in the same genetic (twin colonies) and statistically robust environment.

From a neurophysiological perspective, *Botryllus* neurons express multiple genes coding for voltage-gated Na^+^ and Ca^2+^ ion channels homologous to mammalian ion channel genes, and they reveal complex firing patterns arising both spontaneously and following stimulation.^9^ Notably, though whether *Botryllus* may have some simple form of learning is still unclear, this small animal displays different very simple behavioural responses depending on the activation of different receptors (e.g. contraction of the oral and atrial siphons, contraction of the body wall muscles and variation in heart beating frequency), thus suggesting that there are several discrete sensory motor circuits,^8^ as occurs in humans and other mammals. More importantly, recent research has revealed that this colonial invertebrate expresses a high number of significant genes codifying for proteins involved in human neurodegeneration. Significantly, these genes are differentially expressed in different life phases.^8^ Moreover, degenerating neurons exhibit morphological features and multiple cell death pathways as evidenced in human neurodegenerative diseases, thus paralleling human proteinopenias. Indeed, amyloidogenesis, whose dysregulation causes in human the amyloid deposition provoking neuronal death in Alzheimer’s disease,^5^ is physiologically active in *Botryllus*.^10^ It is to note that in the same colony, developing and degenerating individuals share the same vascular system. This means that, whereas they are all exposed to the same circulating factors, growing buds (with their developing brains) are not affected, i.e. in some extent they are protected, by the degeneration of their parents. These peculiar features may suggest a prion-like mechanism driving the physiological neurodegeneration in *Botryllus*.^11^ This insight aligns with proteinopenias as current hypotheses on human neurodegenerative diseases,^12,13^ opening a new scenario on both their evolution and possible mechanisms of neuroprotection.

As the budding process occurs repeatedly in the colony, giving it continuous new chances of surviving, this little animal may not only conceal the key to the elixir of life but also unlocks an ancient secret about cell proliferation and tumours. Under favourable conditions in nature, colonies grow up indefinitely, as for one degenerating zooid, one or more younger buds replace it.^14^ A circle of life closely resembling what described in Genesis 3:19: ‘for dust you are, and to dust you will return’. Interestingly, senescence can be delayed *in vitro* by extirpating buds from adult individuals, confirming the intimate relationship between death and life.^15^ In *Botryllus*, stem cells, both belonging to the somatic and the germ line, can repeatedly circulate into the vasculature of the colony, invading the stem cell niches of the forming buds, thus assuring the rejuvenation colonial potential. Therefore, questions regarding cell proliferation and cancer may find in *Botryllus* unexpected answers. Nevertheless, aging also occurs in the colony as a whole: in the Lagoon of Venice, typically colonies live a couple of years, but in a laboratory on the coast of central California, where biomedical researchers at Stanford University’s Hopkins Marine Station have used the animal as a model for stem cell biology, some colonies were maintained also for 20 years. Surprisingly, also individuals of old colonies differentially express genes associated to neurodegenerative diseases with respect to those of young colonies (e.g. superoxide dismutase-1; presenilin-1; amyloid precursor protein; Mitogen activated protein kinase-1), other than impaired behavioural performances and fewer neurons. Among those genes, some are in common with the ones expressed in the cyclical, weekly neurodegeneration; others are specifically associated with colony aging.^8^

While *Botryllus* has been the subject of zoological studies for decades, it remains largely unknown to clinical neurologists and neuroscientists as a potential disease model for common neurological disorders. Compared to other animal models, *Botryllus* is easily available and cheap, and experiments do not imply any ethical concern or statement. Converging genetic, evolutionary and neurophysiological evidence supports a link between this simple marine organism and basic mechanisms of human neurodegeneration. Based on these findings, *Botryllus* may represent a new, fascinating model for development and regeneration, with the potential to reveal mechanisms of human disorders, including Alzheimer’s disease and Parkinson’s disease. This may lead to the identification of novel pharmacological targets and the development of innovative non-pharmacological strategies—given the short life cycle of this simple and small animal—in a relatively short time.
